# Supplementary material for: A naturally occurring epiallele associates with leaf senescence and local climate adaptation in Arabidopsis accessions
Source: Nat Commun. 2018 Jan 31;9:460. doi: 10.1038/s41467-018-02839-3 (PMC5792623; doi:10.1038/s41467-018-02839-3)
Supplement: Supplementary file 1 — Supplementary Information [file 41467_2018_2839_MOESM1_ESM.pdf]

A Naturally Occurring Epiallele associates with Leaf Senescence and Local Climate Adaptation in *Arabidopsis* accessions

He et al.

Supplementary Notes

Origin of NMR19 elements

Because there are two copies of NMR19 (NMR19-16 and NMR19-4) in the genome of different *Arabidopsis* accessions, we supposed that both copies were present in the common ancestor of *Arabidopsis thaliana* and we found three pieces of evidence to support that this is indeed the case. 1) NRM19-16 and NMR19-4, if present, are located at the same genomic context in all accessions examined (Supplementary Fig. 3), which unlikely resulted from independent transpositions; 2) The presence and absence patterns of the two NMR19 copies in the phylogenetic tree of all examined accessions indicate that neither copy emerged in a particular sub-clade. (Supplementary Fig. 6); 3) Estimation of the insertion time of NMR19-4 indicates that it was inserted to the genome 0.37-0.98 million years ago, after the divergence of *A. lyrata* and *A. thaliana*, but before the separation of different *A. thaliana* accessions. (Fig. 7d). To address if NMR19-4 (truncated LINE1) originated from retrotransposition of NMR19-16, we blasted the sequence of NMR19-16 against TAIR10 reference genome, and *A.lyrata* and *C.rubella* genome. We only found one LINE1 retrotranposon of 600 bp, AT2TE01560, with high sequence homology to NMR19-16 in the TAIR10 reference genome. It is unlikely that the longer element NMR19-4 (2500 bp) resulted from retrotransposition of the shorter element AT2TE01560 (600 bp). In addition, NMR19-16 and NMR19-4 showed a much closer relationship (~98% sequence similarity) than other NMR19 homologs (Supplementary Fig. 12c). Moreover, NMR19-16 contains all the necessary components for retrotransposition of LINE1 (Fig. 2e) to produce NMR19-4. Altogether, we concluded that NMR19-4 (truncated LINE1) originated from retrotransposition of NMR19-16 (full length LINE1).

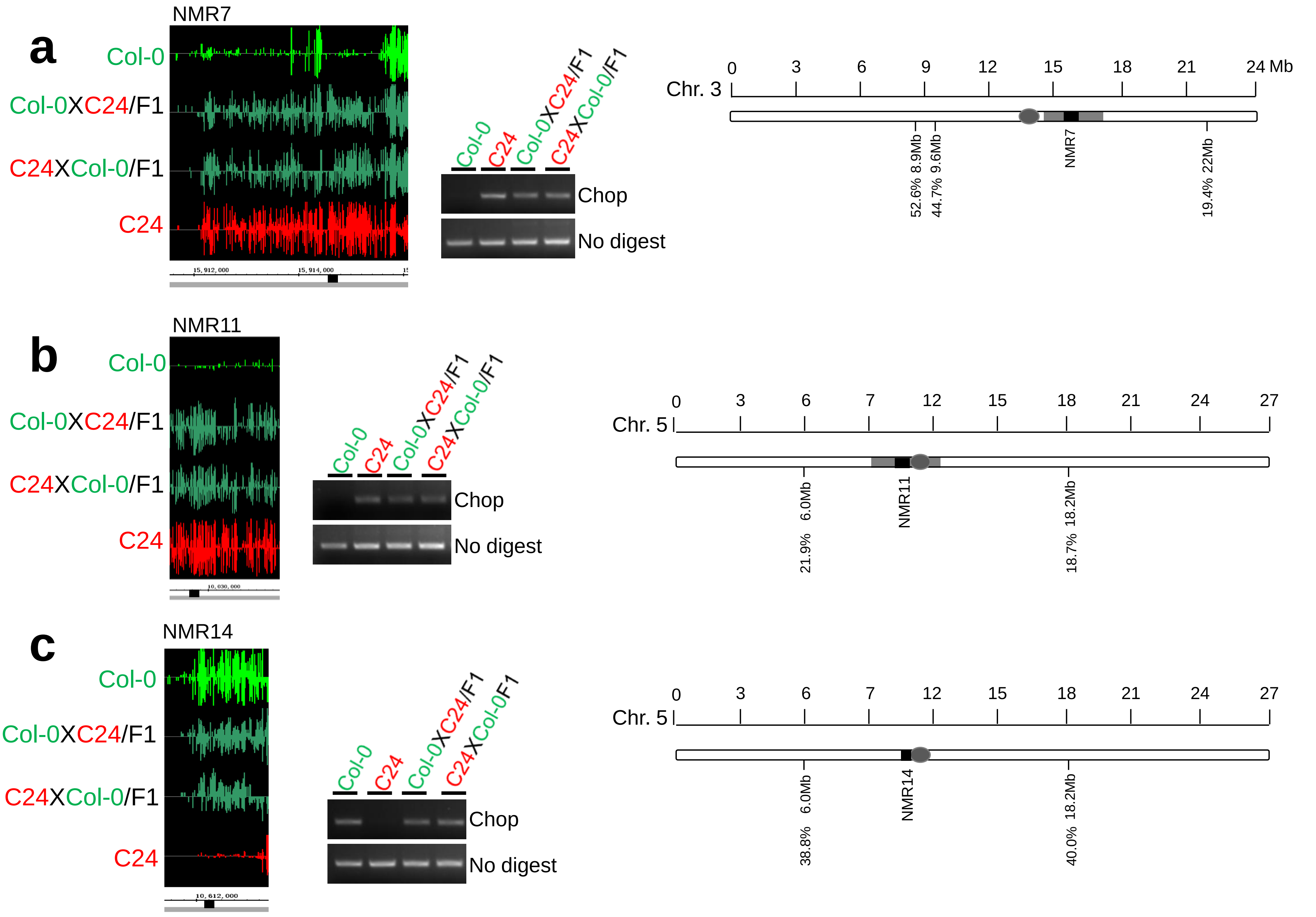

Supplementary Figure 1 Linkage analysis of _n_aturally occurring DNA _m_ethylation variation _r_egions (NMRs). Left, IGB snapshot of NMRs in Col-0, Col-0XC24/F1, C24XCol-0/F1, and C24 from BS-seq data, The black bar indicates enzyme cutting position for Chop-PCR; Middle, Chop-PCR validation of the NMR19; Right, recombination rate between indicated position and methylation status of NMRs, black bar indicates location of NMRs, gray rectangle represents predicated linked region, and gray oval indicates centromere. We first selected 22 NMRs (NMR1 to NMR 24) from 10,581 differentially methylated regions generated by genome-wide analysis. We then examined the 22 NMRs through Chop-PCR assays and chose 7 validated NMRs for genetic analysis (**Supplementary Data 1**). Four of 7 NMRs were used in the linkage analysis.

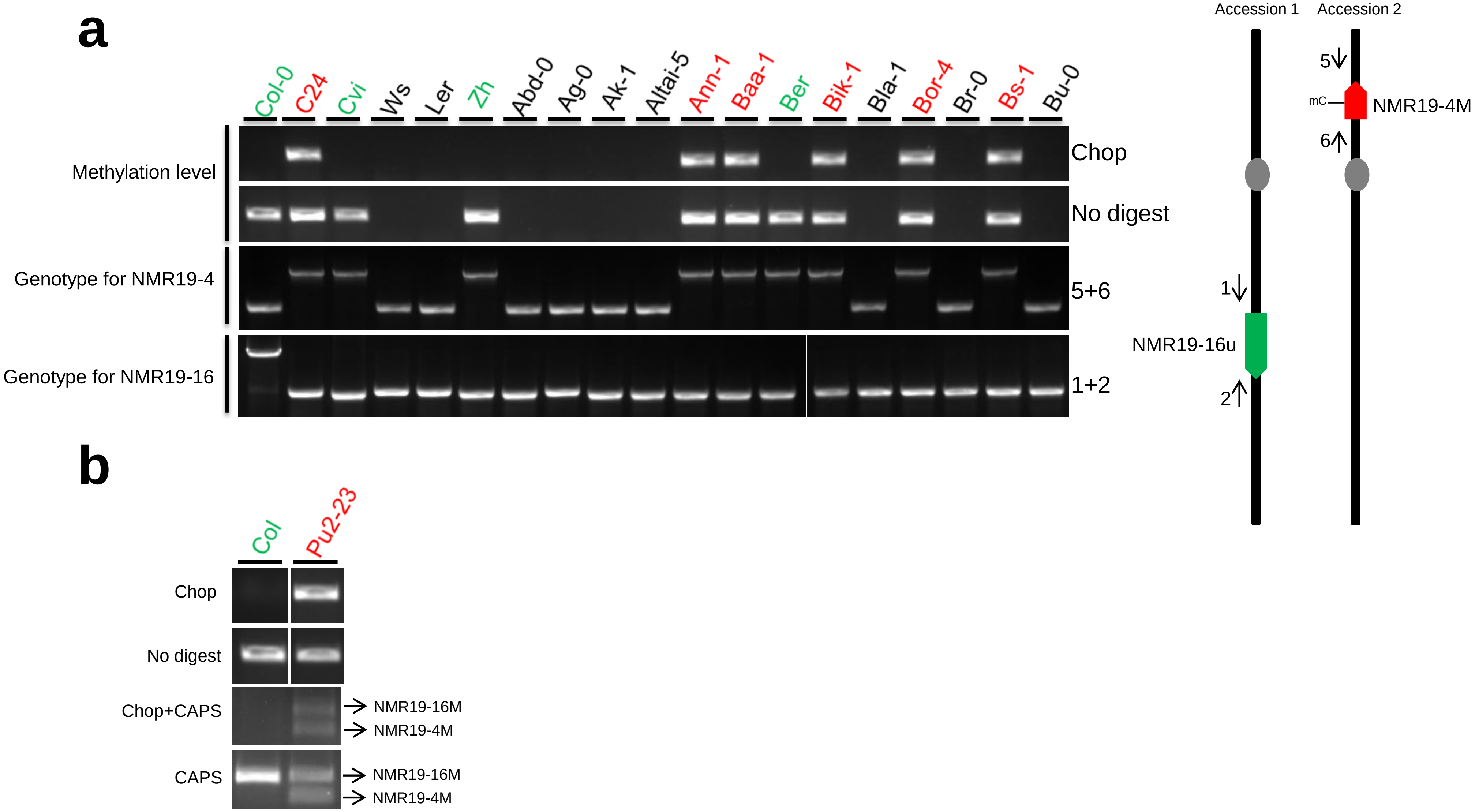

Supplementary Figure 2 Identification of five types of NMR19 through the genotyping of 140 accessions.
(**a**) Example of genotyping of methylation levels and location of NMR19. Left, results of Chop-PCR and PCR assays; Right, schematic representation of NMR19, the numbered arrows indicate the primers used in (left).
(**b**) Allele-specific Chop-PCR result in the Pu2-23 accession. As shown, Pu2-23 contains NMR19-16m and NMR19-4m.

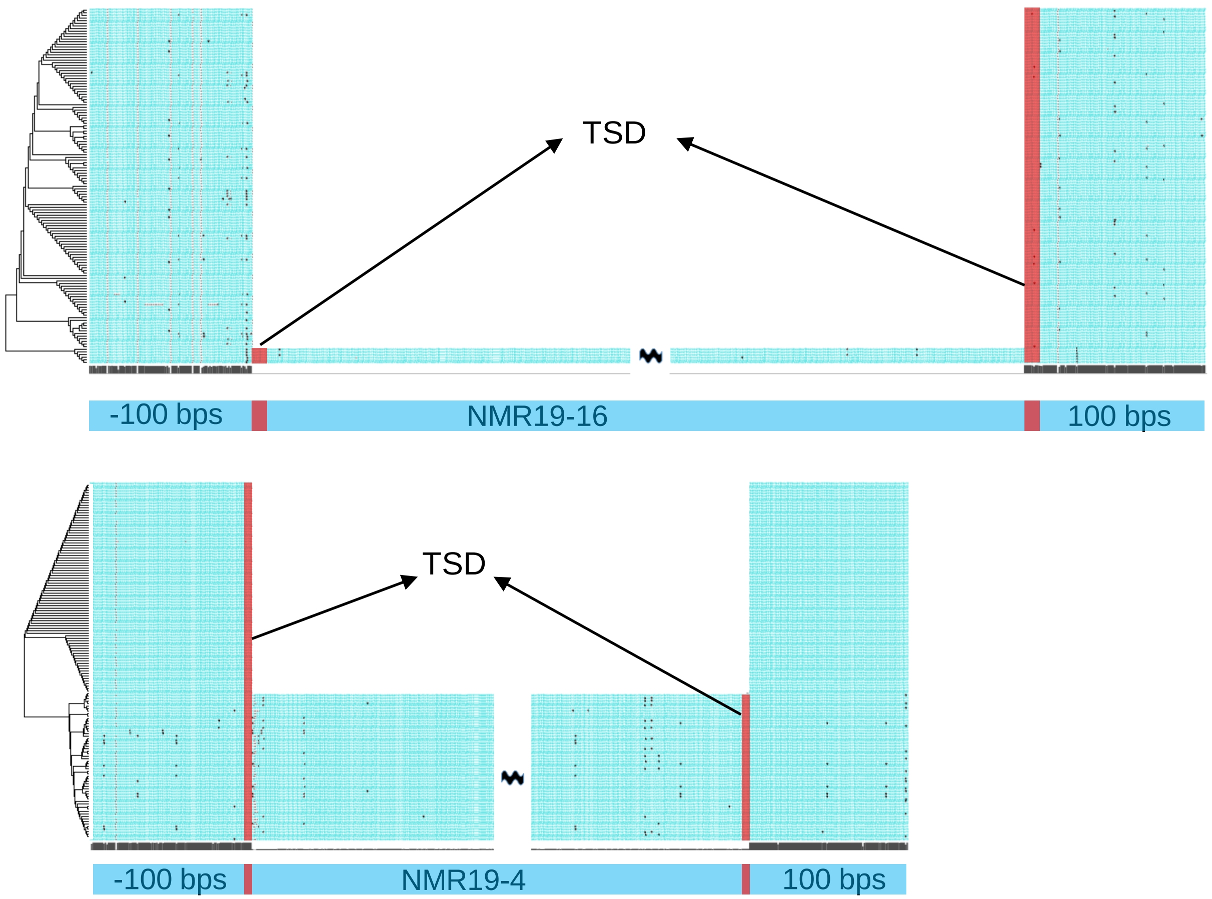

TSD: target site duplication

Supplementary Figure 3 Alignment of NMR19 sequences together with flanking sequences in 140 accessions.
For better display, middle parts of NMR19-16 (upper, black tilde) and NMR19-4 (bottom, black tilde) were omitted.

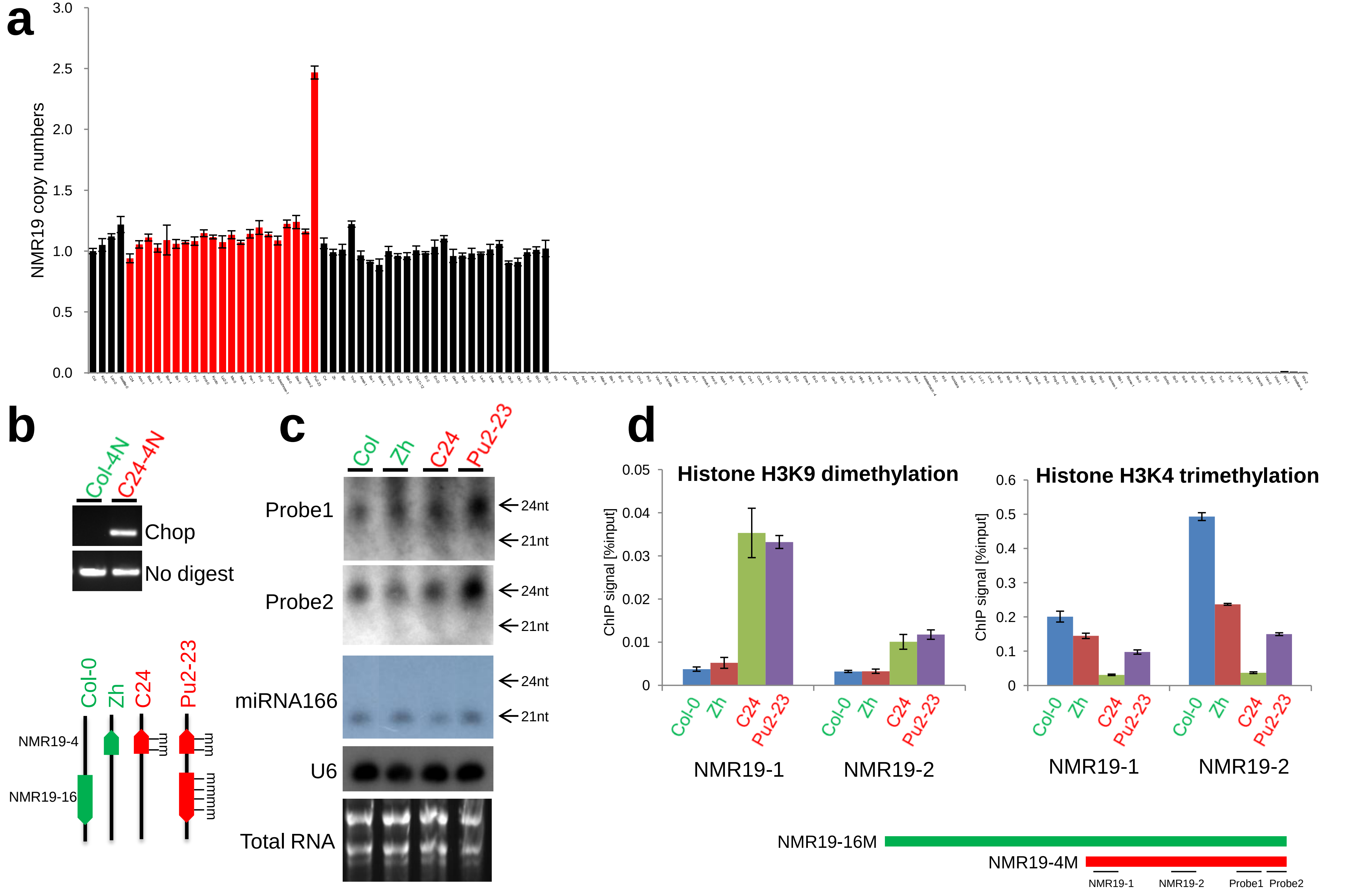

Supplementary Figure 4 Methylation variation of NMR19 is associated with chromatin state in *Arabidopsis thaliana* accessions.

(**a**) Estimation of NMR19 copy numbers in 132 accessions. DXR (1-Deoxy-d-xylulose 5-phosphate reductoisomerase), which is a single-copy gene, was used as the internal control. Red, accessions with methylated NMR19; Black, accessions with unmethylated NMR19. Error bars are defined as s.e.m (n=3).

(**b**) Methylation levels of NMR19-4 and NMR19-16 in tetraploid Col-0 and C24 accessions. The result shows that the methylation variation of NMR19 is not associated with the copy number.

(**c**) RNA blot analysis of NMR19 siRNA levels in Col-0, C24, Zh, and Pu2-23 accessions. The probe is shown in bottom of (**d**).

(**d**) Histone H3K9me2 (left) and H3K4me3 (right) levels at NMR19 in Col-0, Zh, C24, and Pu2-23 accessions, as determined by chromatin immunoprecipitation assay. Data were normalized by input DNA. The region of q-PCR is indicated in the bottom. Error bars are defined as s.e.m (n=3).

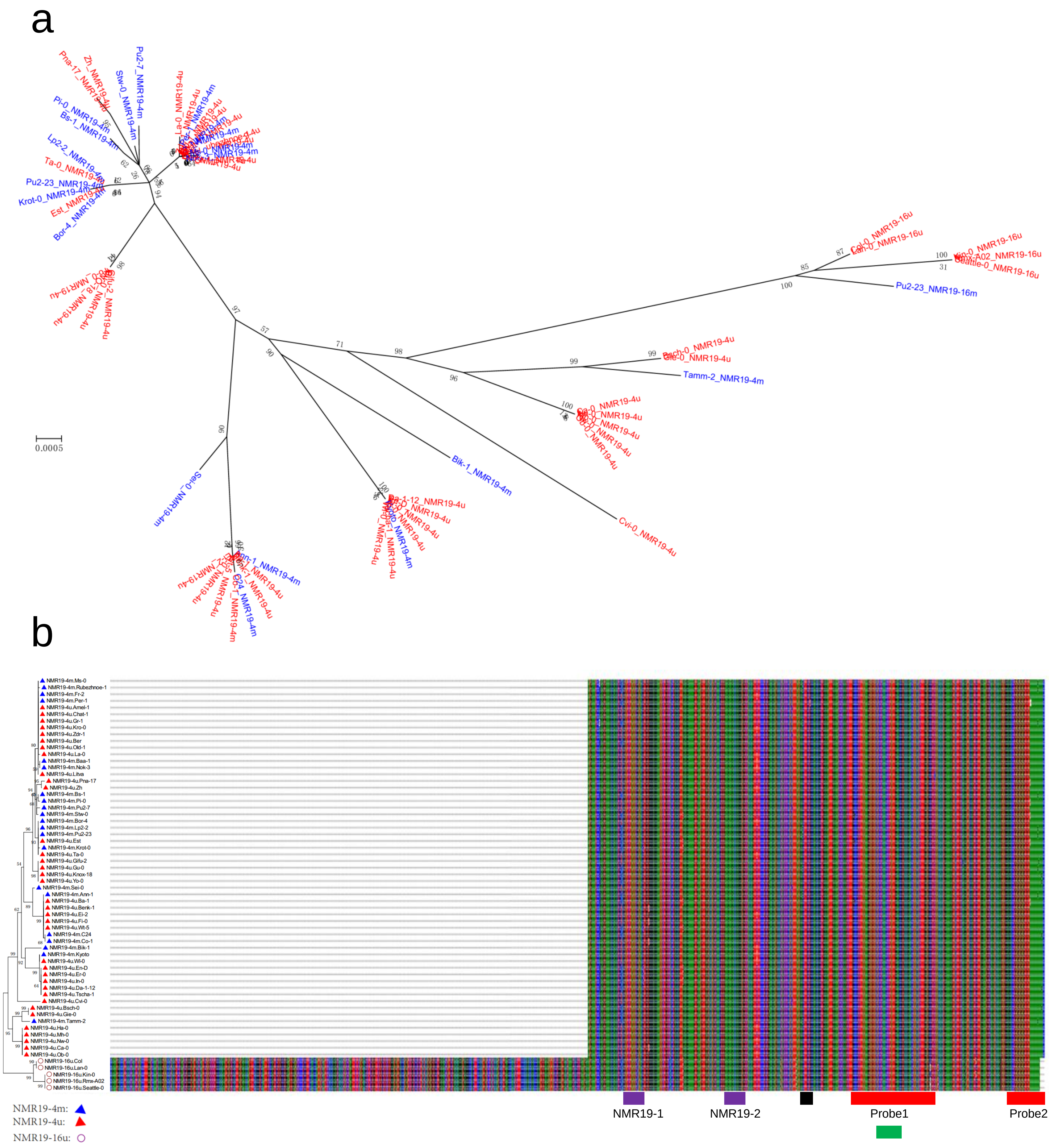

Supplementary Figure 5 Phylogenetic analysis of NMR19 sequences identified in different accessions.

(**a**) The evolutionary history was inferred by using the Maximum Likelihood method based on the Tamura-Nei model[1]. The percentage of trees in which the associated taxa clustered together (calculated from bootstrap test with 500 replicates) is shown next to the branches. All positions with less than 95% site coverage were eliminated. That is, fewer than 5% alignment gaps, missing data, and ambiguous bases were allowed at any position. There were a total of 2,567 positions in the final dataset. Evolutionary analyses were conducted in MEGA7[2].

(**b**) Alignment of NMR19 sequences. The black bar indicates enzyme cutting position for Chop-PCR; purple bar indicates the region used for ChIP-qPCR; red bar indicates the region used for small RNA Northern blot assay; green bar indicates the region used for copy number analysis.

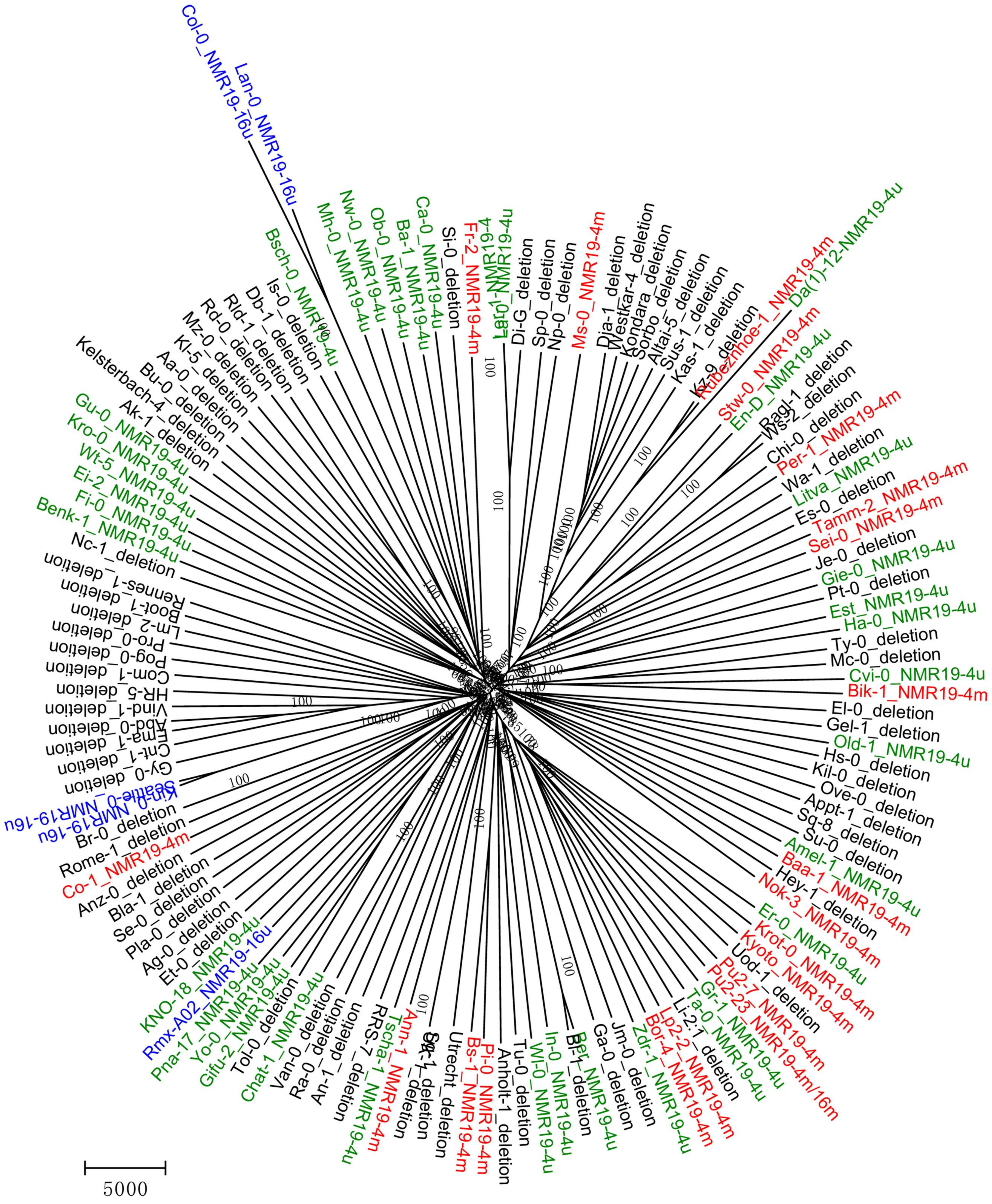

Supplementary Figure 6 Phylogenetic tree of *Arabidopsis thaliana* accessions using genome-wide single nucleotide polymorphism data.
The evolutionary history was inferred using the Neighbor-Joining method [3] implemented in MEGA7[2]. The percentage of replicate trees in which the associated taxa clustered together (calculated from bootstrap test with 500 replicates) are shown next to the branches[4]. The analysis involved 137 nucleotide sequences. All ambiguous positions were removed for each sequence pair. A total of 100,000 SNP positions for 137 *A. thaliana* accessions were extracted from the 1001 Genomes Project and used for phylogenetic analysis[5]. The presence/absence and the methylation status of NMR copies are annotated after the name of each accession.

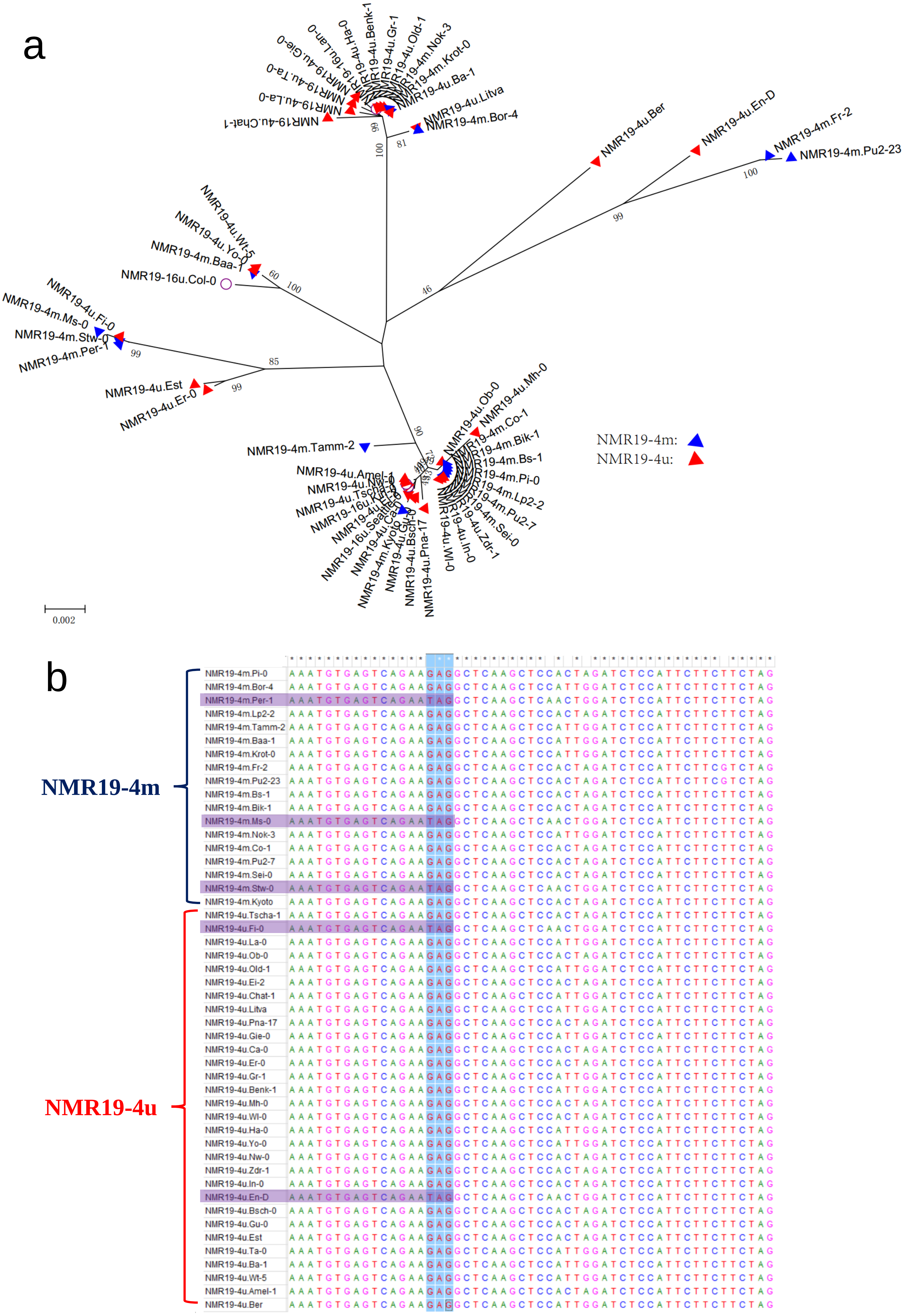

Supplementary Figure 7 Phylogenetic relationships of *CMT2* CDS (Coding sequence) between *A. thaliana* accessions with NMR19-4m and with NMR19-4u.
(**a**) Phylogenetic analysis of *CMT2* CDS sequences in *A. thaliana* accessions with NMR19-4.
(**b**) Alignment of partial sequences of the first exon of *CMT2* in *A. thaliana* accessions with NMR19-4.
The highlighted in purple indicate the accessions with $CMT2_{stop}$ alleles (Shen et al. 2014 Plos Genet).

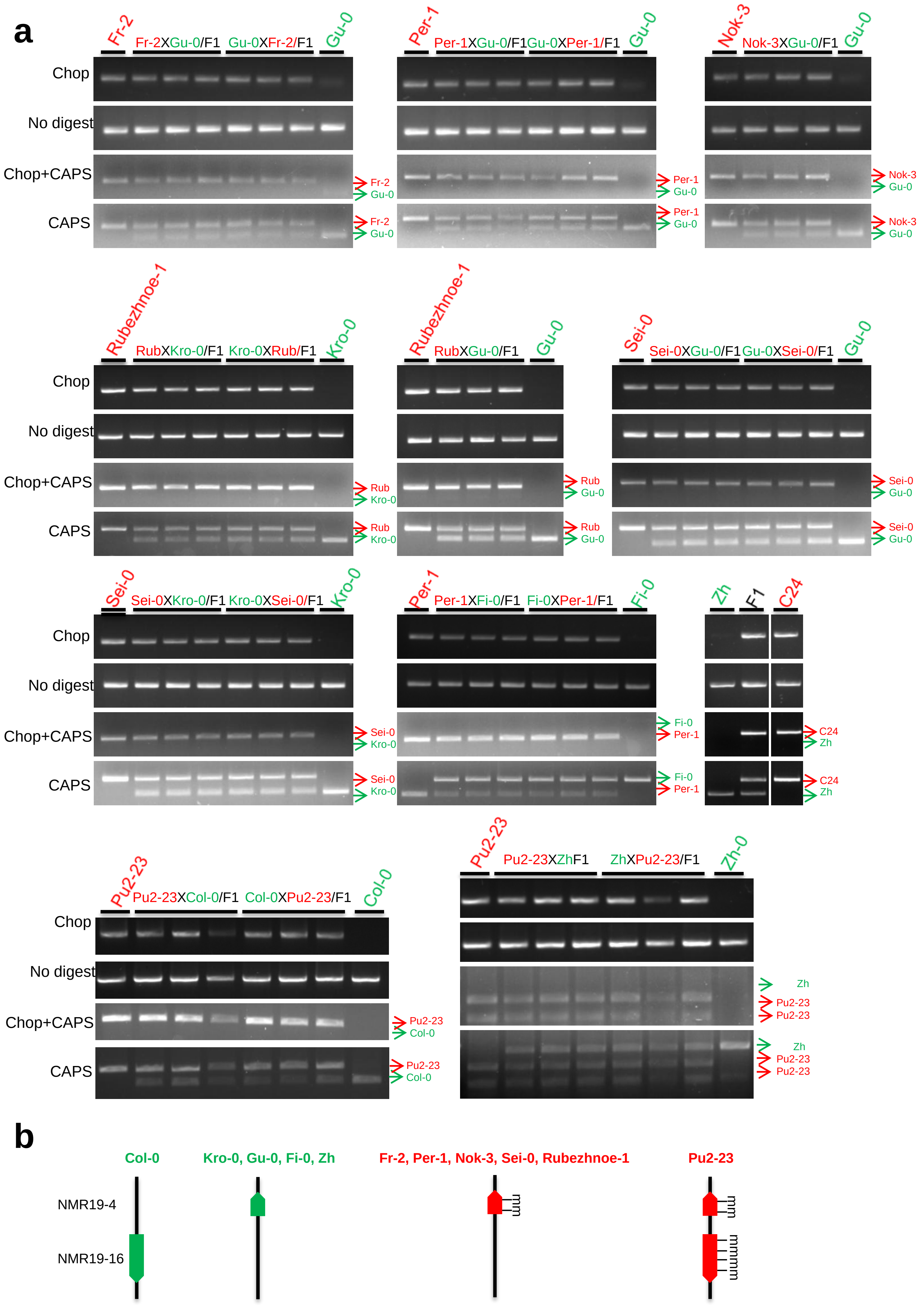

Supplementary Figure 8 Allele methylation pattern of NMR19 in F1 progeny of crosses between different parental lines.

(**a**) Examination of allele-specific methylation status based on CAPS-PCR (Cleaved Amplified Polymorphic Sequences PCR) and methylation-sensitive PCR in the F1 progeny. The arrows on the right of gel indicate the alleles.

(**b**) Schematic representation of the presence/absence and the methylation status of NMR19 in different accessions, which were used for the assays in (**a**). m indicates methylated NMR19.

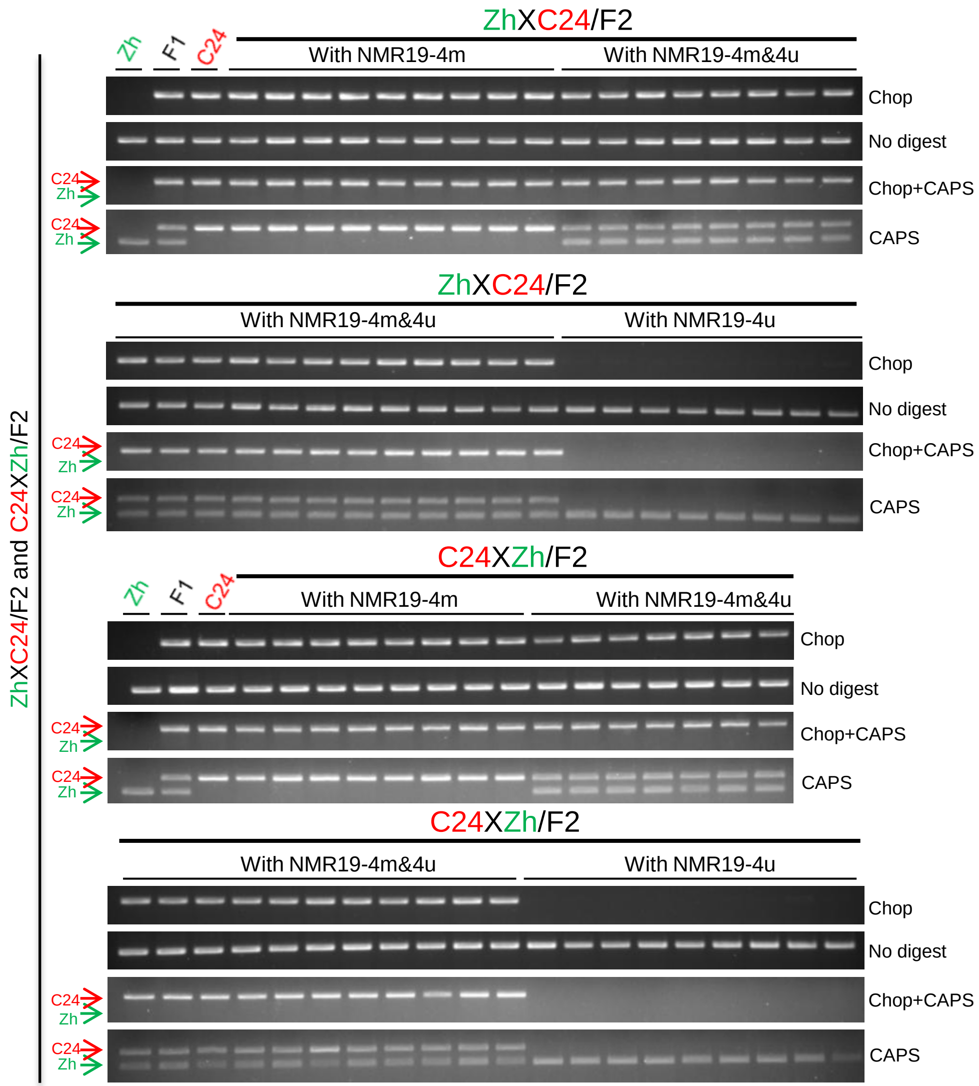

Supplementary Figure 9 Allele methylation pattern of NMR19-4 in F2 derived from a C24 and Zh cross. Examination of allele-specific methylation status based on CAPS-PCR (Cleaved Amplified Polymorphic Sequences PCR) and methylation-sensitive PCR in F2 progeny. The arrows on the left indicate the alleles corresponding to the bands on the gel.

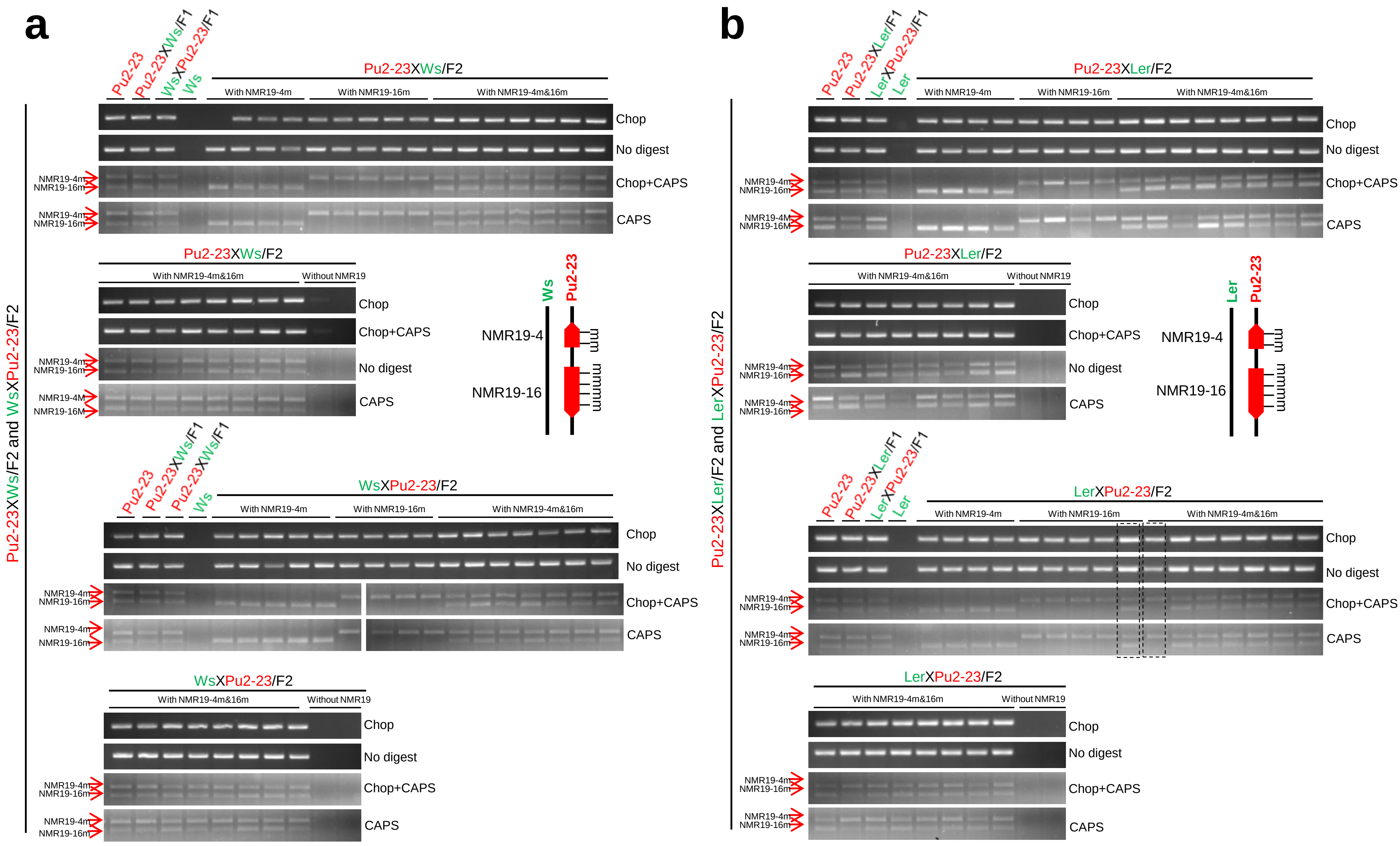

Supplementary Figure 10 Allele methylation pattern of NMR19-4 and NMR19-16 in F2 derived from crosses between Ws and Pu2-23 (**a**) and between Ler and Pu2-23 (**b**).The arrows on the left indicate the alleles corresponding to the bands on the gel.

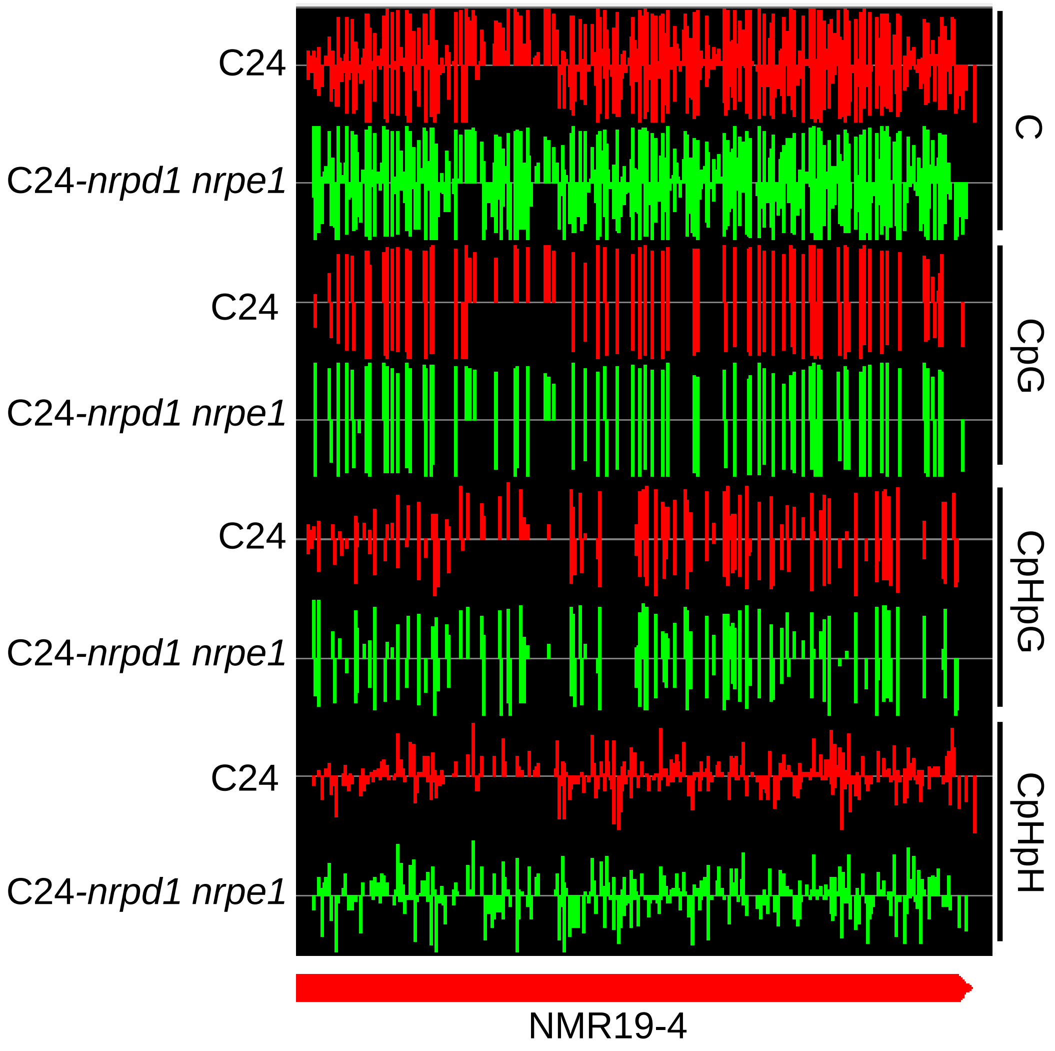

Supplementary Figure 11 Methylation profile of NMR19-4 in wild type C24 and C24-*nrpd1 nrpe1* double mutant. Shown are IGB snapshots of NMR19-4 methylation from BS-seq data.

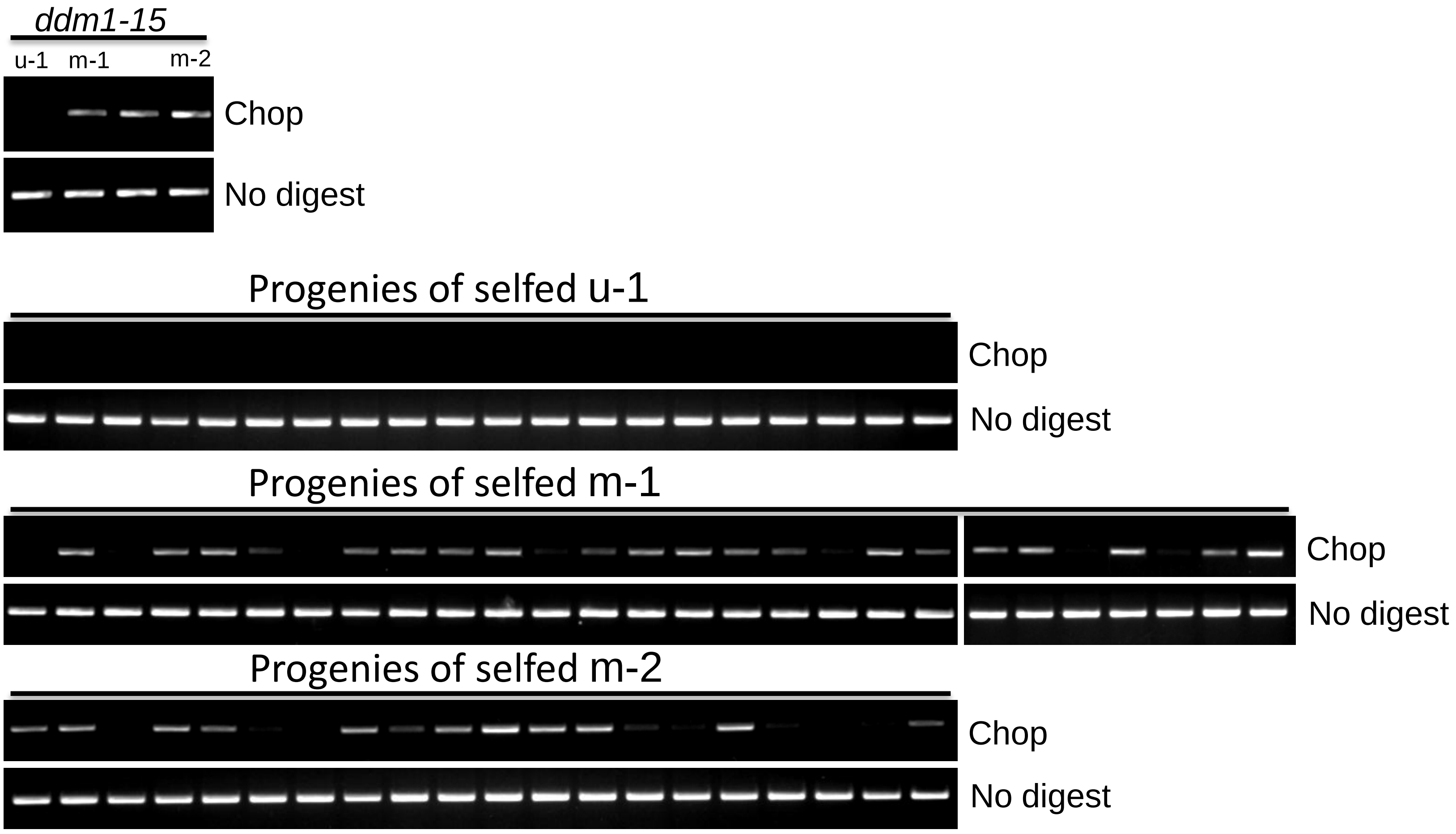

Supplementary Figure 12 Methylation status of NMR19-4 in *ddm1-15* inbred lines.

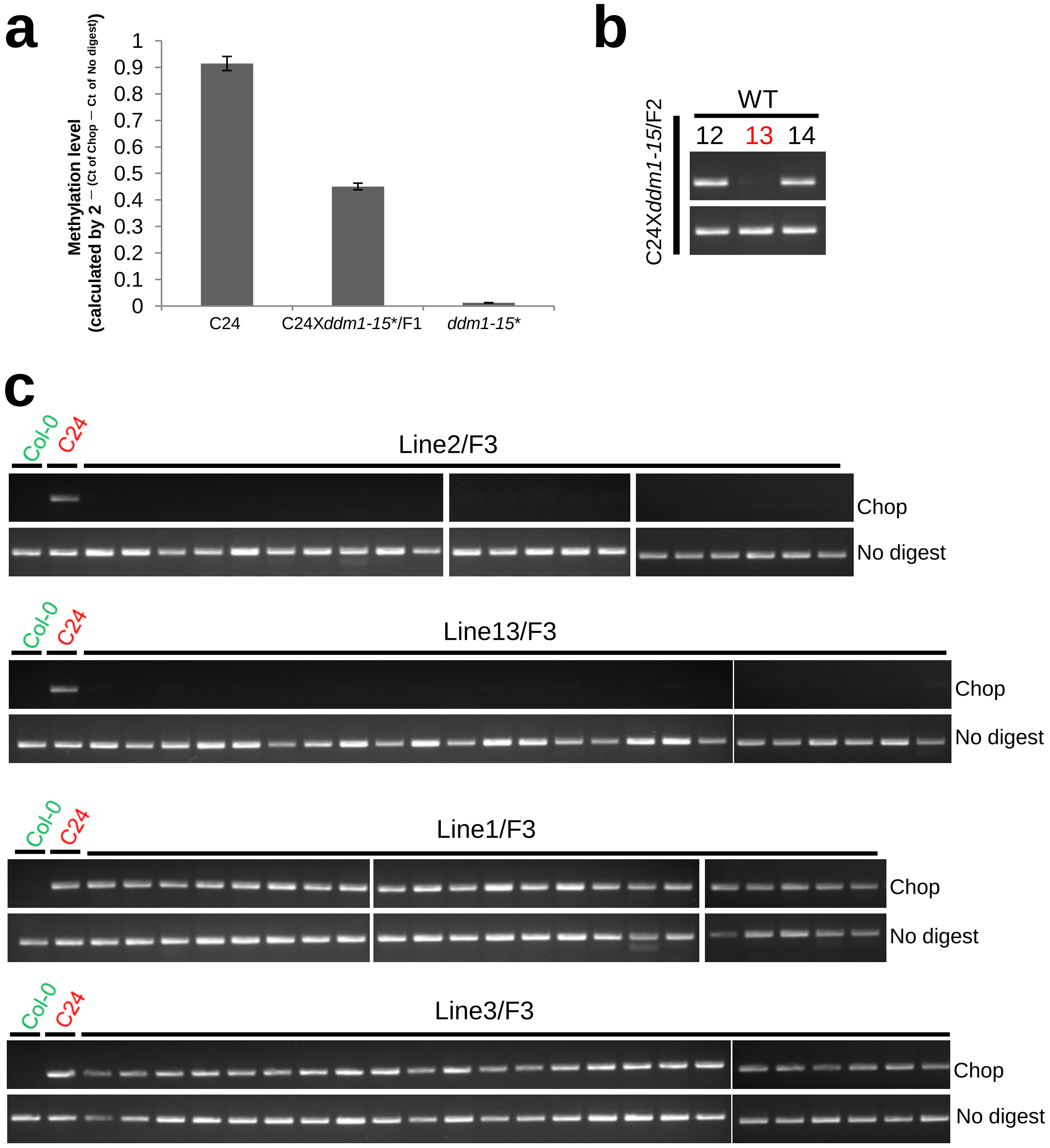

Supplementary Figure 13 Methylation status of NMR19-4 was maintained in F3 (related to Fig. 4b).

(**a**) DNA methylation levels of NMR19-4 in F1 of backcross determined by restriction enzyme digestion and q-PCR. Error bars are defined as s.e.m (n=3).
(**b**) Methylation pattern of NMR19-4 in F2 progenies of a backcross. WT, the F2 individual plant without *ddm1* mutation; the red colored numbers indicate the offspring of this line used for F3 analysis in (**b**).
(**c**) Methylation status of NMR19-4 was maintained in F3. F3 progenies were derived from the F2 lines that are shown in (**a**) and (**Fig. 4b**).

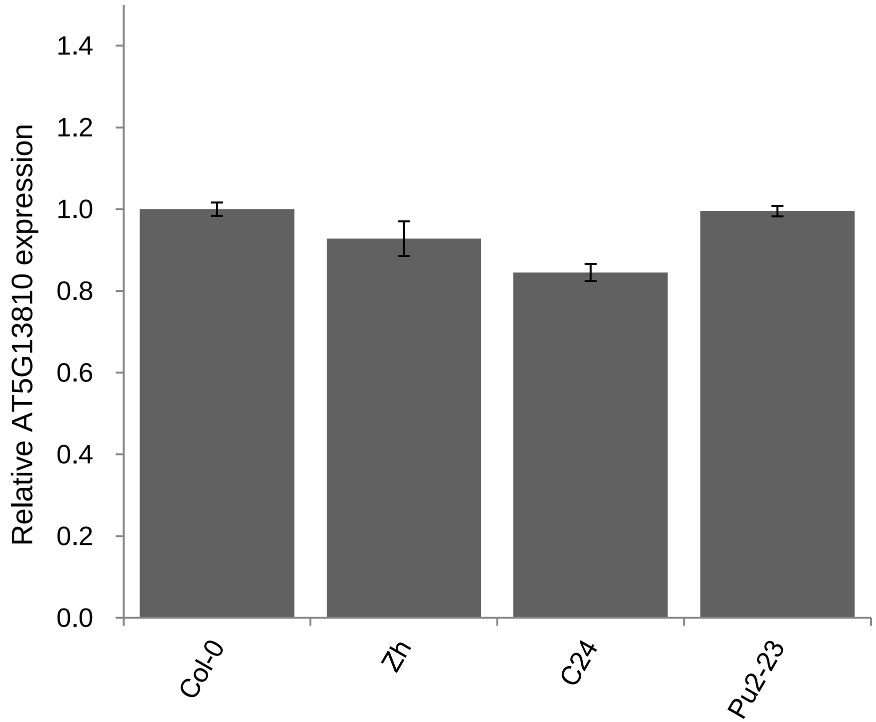

Supplementary Figure 14 Expression levels of AT5G13810 gene in different Arabidopsis accessions. Error bars are defined as s.e.m (n=3).

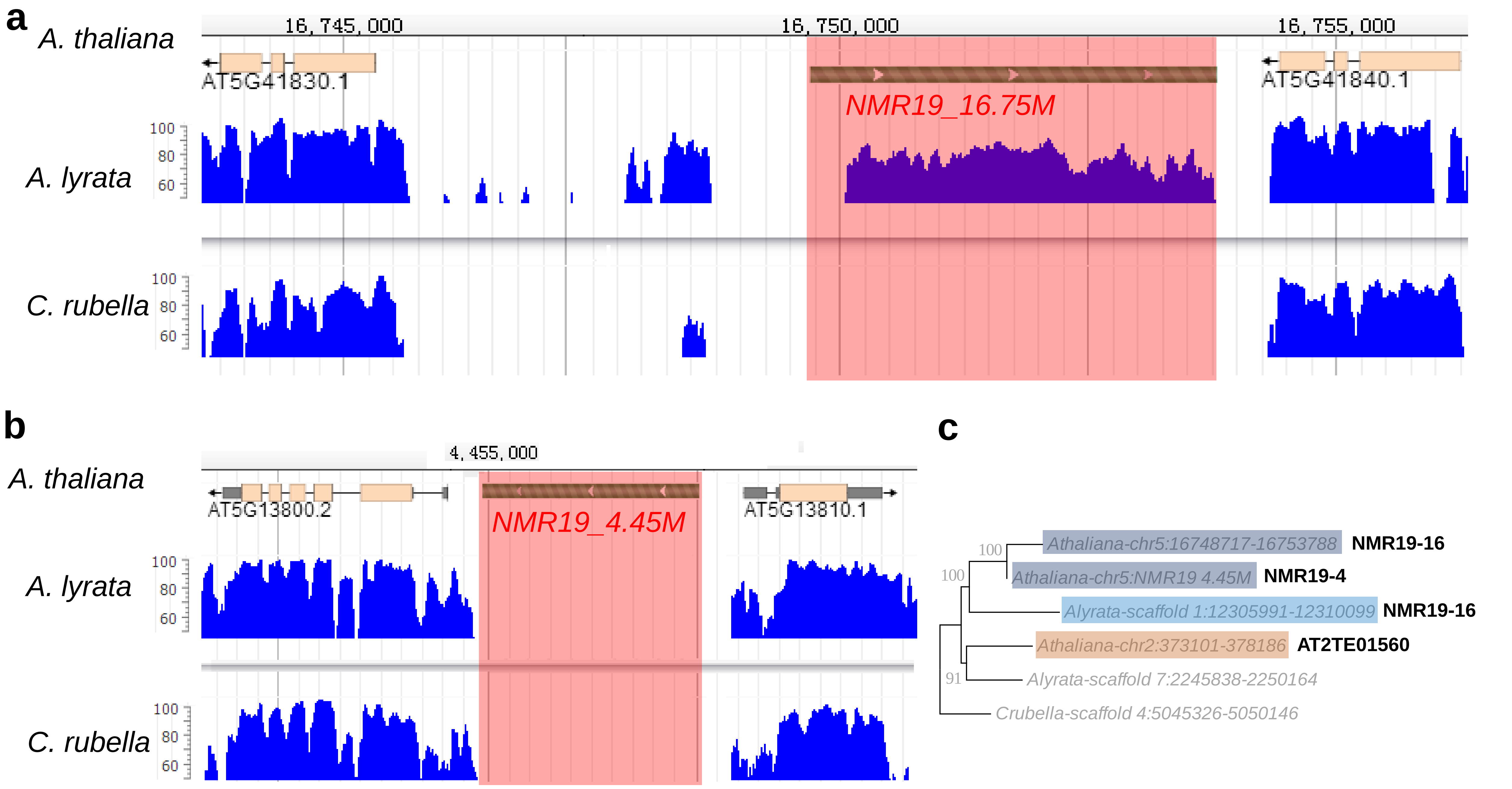

Supplementary Figure 15 NMR19-16 is conserved in *A.thaliana* and *A.lyrata*.
Flanking genes of NMR19-16 (**a**) or (**b**) NMR19-4 are conserved in *A.thaliana*, *A.lyrata* and *C.rubella*, indicating that NMR19-16 and NMR19-4 reside in the same genomic locations and that their emergence most likely resulted from a single insertion event before the divergence of these species. Highlighted pink region indicates the position of NMR19. (**c**) Phylogenetic tree of NMR19 homologs in *A.thaliana*, *A.lyrata* and *C.rubella*. *A.thaliana* NMR19-4 and NMR19-16 sequences were Blasted against the genome sequences of *A.thaliana*, *A.lyrata* and *C.rubella* to obtain NMR19 homologs and phylogenetic tree was constructed by MEGA7[2].

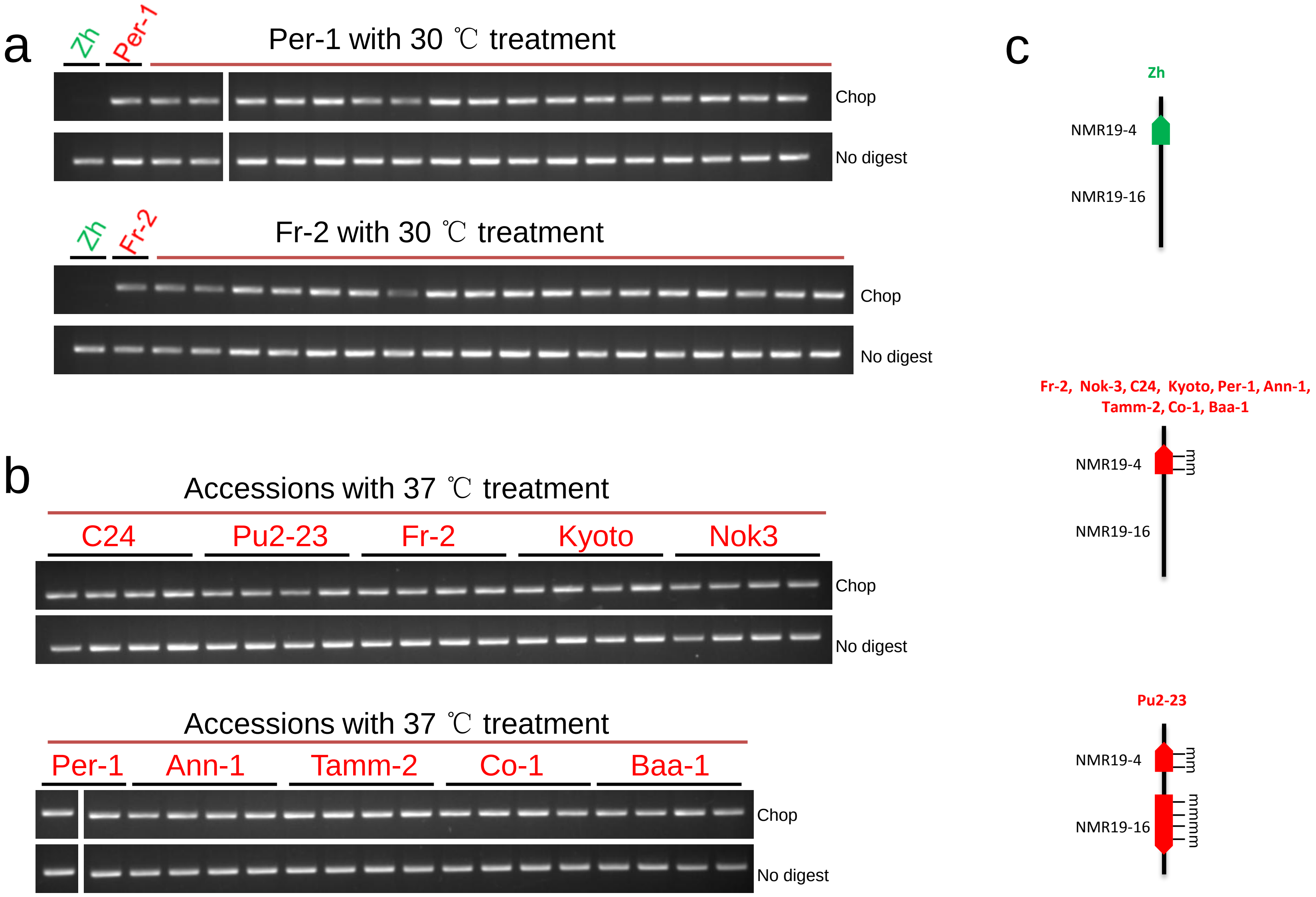

Supplementary Figure 16 Examination of DNA methylation levels in different *Arabidopsis* accessions after high temperature treatment.
Accessions were subjected to 30 ℃ (**a**) or 37 ℃ heat treatment (**b**), showed no obvious alternations in the methylation level after high temperature treatment. (**c**) Schematic representation of the methylation status of NMR19 in the accessions used in the heat stress assays. m indicates methylated NMR19.

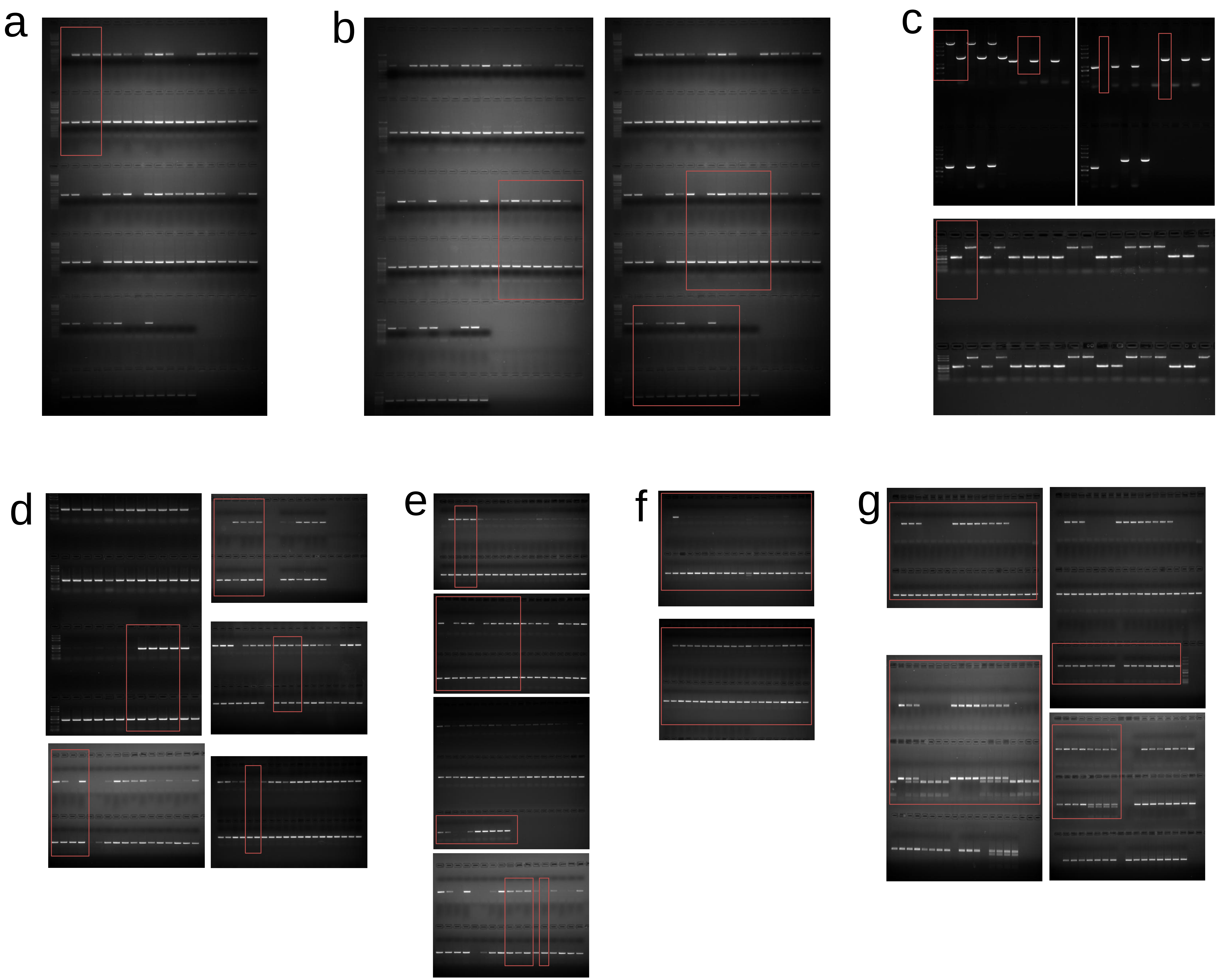

Supplementary Figure 17 Uncropped gels/blots were used in figures. Related lanes are marked with red rectangle. (**a**) for Fig. 1a, (**b**) for Fig. 1b, (**c**) for Fig. 2d, (**d**) for Fig. 4a, (**e**) for Fig. 4b, (**f**) for Fig. 4c, (**g**) for Fig. 6a.

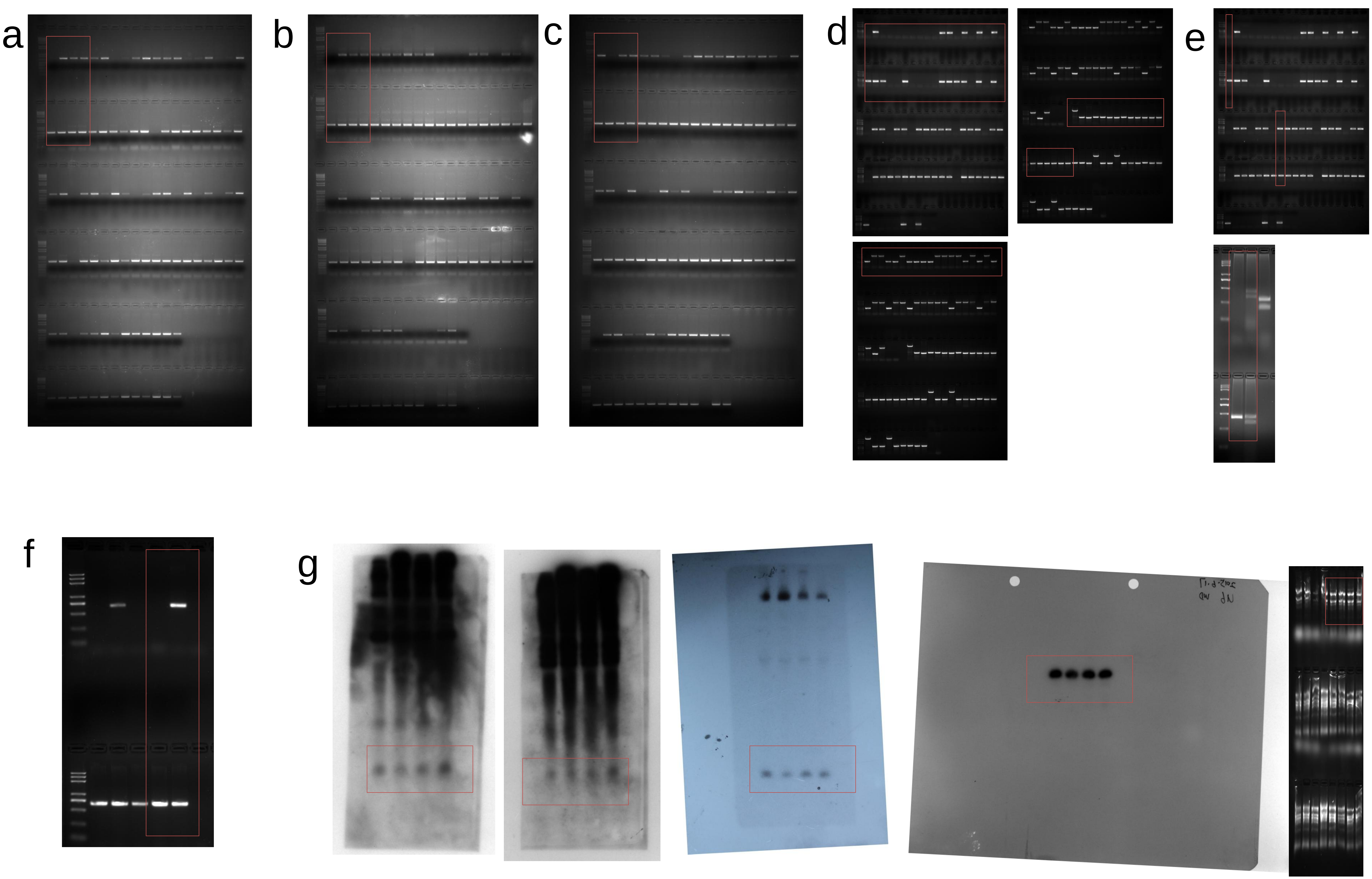

Supplementary Figure 18 Uncropped gels/blots were used in supplementary figures. Related lanes are marked with red rectangle. (**a**) for Supplementary Fig. 1a, (**b**) for Supplementary Fig. 1b, (**c**) for Supplementary Fig. 1c, (**d**) for Supplementary Fig. 2a, (**e**) for Supplementary Fig. 2b, (**f**) for Supplementary Fig. 4b, (**g**) for Supplementary Fig. 4c.

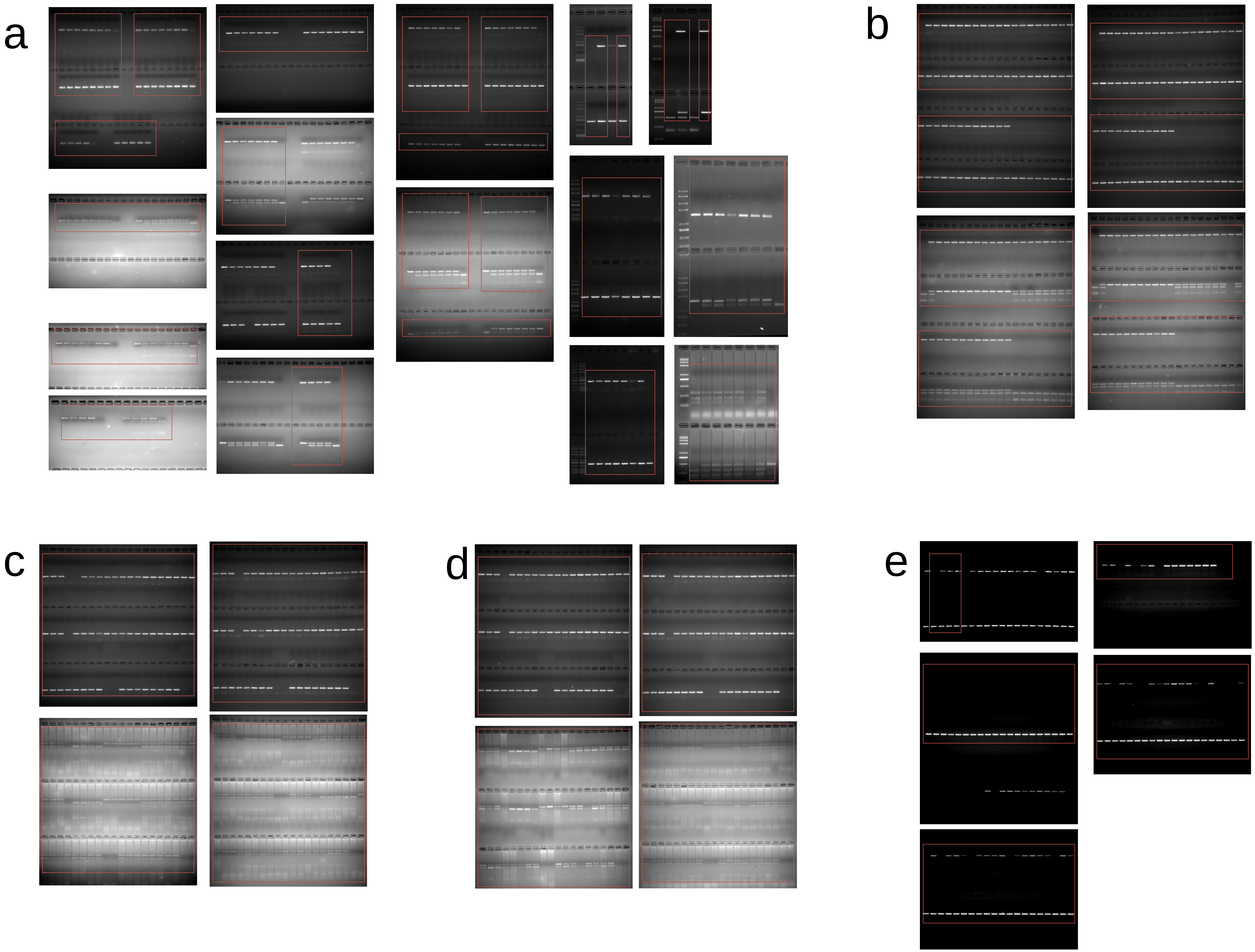

Supplementary Figure 19 Uncropped gels/blots were used in supplementary figures. Related lanes are marked with red rectangle. (**a**) for Supplementary Fig. 8a, (**b**) for Supplementary Fig. 9, (**c**) for Supplementary Fig. 10a, (**d**) for Supplementary Fig. 10b, (**e**) for Supplementary Fig. 12.

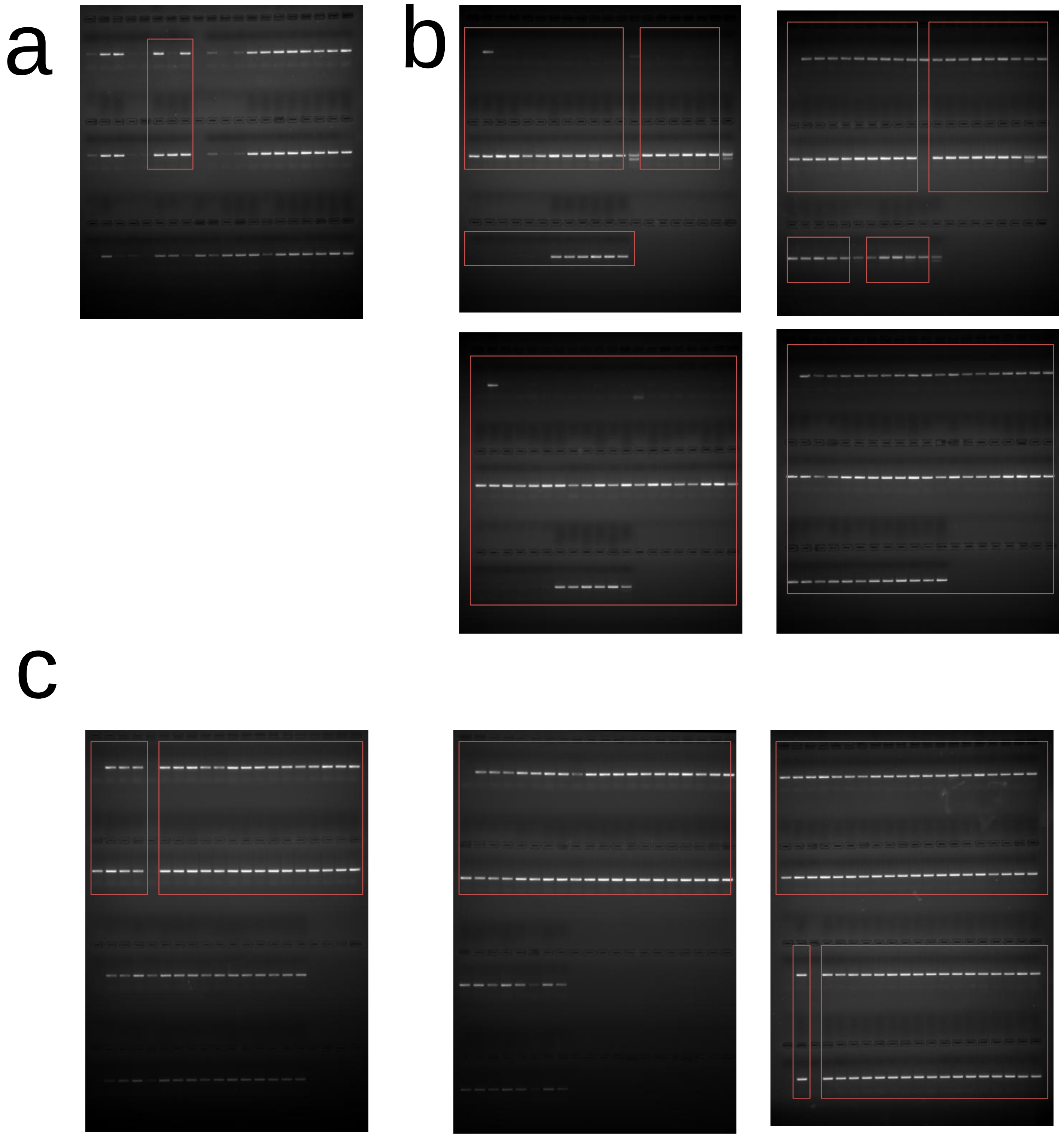

Supplementary Figure 20 Uncropped gels/blots were used in supplementary figures. Related lanes are marked with red rectangle. (**a**) for Supplementary Fig. 13b, (**b**) for Supplementary Fig. 13c, (**c**) for Supplementary Fig. 16.

Supplementary References

1. Tamura, K. & Nei, M. Estimation of the number of nucleotide substitutions in the control region of mitochondrial DNA in humans and chimpanzees. *Mol Biol Evol* **10**, 512-26 (1993).

2. Kumar, S., Stecher, G. & Tamura, K. MEGA7: Molecular Evolutionary Genetics Analysis Version 7.0 for Bigger Datasets. *Mol Biol Evol* **33**, 1870-4 (2016).

3. Saitou, N. & Nei, M. The neighbor-joining method: a new method for reconstructing phylogenetic trees. *Mol Biol Evol* **4**, 406-25 (1987).

4. Felsenstein J. Confidence limits on phylogenies: An approach using the bootstrap. Evolution **39**:783-791 (1985).

5. Consortium, G. 1,135 Genomes Reveal the Global Pattern of Polymorphism in Arabidopsis thaliana. *Cell* **166**, 481-91 (2016).
