# Supplementary material for: A naturally occurring epiallele associates with leaf senescence and local climate adaptation in Arabidopsis accessions
Source: Nat Commun. 2018 Jan 31;9:460. doi: 10.1038/s41467-018-02839-3 (PMC5792623; doi:10.1038/s41467-018-02839-3)
Supplement: Supplementary file 3 — Description of Additional Supplementary Files [file 41467_2018_2839_MOESM3_ESM.pdf]

Description of Additional Supplementary Files

File Name: Supplementary Data 1
Description: Mendelian Inheritance of methylated and unmethylated NMRs

File Name: Supplementary Data 2
Description: Methylation, PPH expression, chlorophyll contents, and climate parameters in different Arabidopsis accessions

File Name: Supplementary Data 3
Description: Correlation coefficient of NMR19-4 methylation, PPH expression, and Chlorophyll contentin Arabidopsis accessions with the climate parameters at the place of origin

File Name: Supplementary Data 4
Description: Primers used in this study

File Name: Supplementary Data 5
Description: Sequence of NMR19 in accessions
